# Effect of physiotherapeutic procedures on the bioelectric activity of the masseter muscle and the range of motion of the temporomandibular joints in the female population with chronic pain: a randomized controlled trial

**DOI:** 10.1186/s12903-023-03601-y

**Published:** 2023-11-25

**Authors:** Magdalena Gębska, Bartosz Dalewski, Łukasz Pałka, Paweł Kiczmer, Łukasz Kołodziej

**Affiliations:** 1 Department of Rehabilitation Musculoskeletal System, Pomeranian Medical University, Szczecin, 70-204 Poland; 2 Department of Dental Prosthetics, Pomeranian Medical University, Szczecin, 70-204 Poland; 3 Orofacial Pain Unit, Pomeranian Medical University, Szczecin, 70-204 Poland; 4 Private Dental Practice, Zary, 68-200 Poland; 5 Department and Chair of Pathomorphology, Faculty of Medical Sciences in Zabrze, Medical University of Silesia, 13-15 3 Maja, Zabrze, 41-800 Poland

**Keywords:** Physiotherapy, Manual therapy, Electromyography, sEMG, Temporomandibular joint, Orofacial pain, Mandible range of motion, TMD

## Abstract

**Introduction:**

Physical therapy (PT) methods applied in dentistry are increasingly discussed nowadays. Taking into account a rapidly growing number of temporomandibular disorders (TMDs) and orofacial pain patients, it is reasonable to determine which of the available physiotherapeutic (PT) methods are more effective than others, especially in terms of their possible analgesic and myorelaxant effects.

**Objective:**

To assess manual and physical factors influencing pain reduction or elimination and increased muscle tension in patients with TMD; yet the influence of the applied forms of PT on the range of motion (ROM) of temporomandibular joints (TMJ).

**Material and methods:**

A randomized, parallel-group, RCT, single-blind, equi-randomized (1:1) study was conducted in DC/TMD Group Ib patients (20–45 years of age). An experimental group (G1, n = 104) and a control group without TMD (G2, n = 104) were created according to CONSORT guidelines. Diagnostic measurements were performed in both groups (mass sEMG, temporomandibular joint range of motion-ROM, pain intensity - NRS). Group G1 was randomly divided (envelope method) into 4 therapeutic groups, in which therapy was carried out for 10 days: magnetostimulation (MS), magnetoledotherapy (MLE), magnetolaserotherapy (MLA), manual therapy (MT). Each time after the therapy, ROM and NRS measurements were performed, and after the 5th and 10th day sEMG.

**Results:**

Statistically significant differences were found in the sEMG values of the masseter muscles, TMJ ROM and the pain intensity in G1 and G2 (p < 0.00). The largest decrease in sEMG (% MVC) of the masseter muscle occurred in the subgroup in which the manual therapy (MT) procedures were applied, p < 0.000. There was no clinically significant difference in and between other subgroups. There was a distinct mandible ROM increase noted in the MT group, with minimal changes in the MLA and MLE groups and no changes in the MS group. There was a clear increase in the lateral mobility of both right and left TMJ in the MT group. There were no differences in the course of the study in the MS group, and slight increases in the MLA and MLE groups. In the case of pain measurements, the greatest decrease in pain intensity was observed in the MT subgroup.

**Conclusions:**

According to our results manual therapy is an effective form of treatment in patients with pain, increased masticatory muscle tension and limitation in mandible ROM. Dental physiotherapy should become an integral part of multimodal TMD patients’ treatment.

## Introduction

Each component of the masticatory system is characterized by a specific structural tolerance, the exceeding of which may lead to tissue damage. Potentially aggravated zones include: masticatory muscles, temporomandibular joints (TMJ), periodontal ligaments and teeth hard tissue [1, 2]. If the weakest structure is muscle tissue, then the patient most often experiences painful sensitivity during the movements of the mandible, a feeling of fatigue, and/or tension in the corresponding orofacial part area [3]. Clinically, this can lead to pathological limited mandibular abduction accompanied by pain. Muscle pain is defined as myalgia and its’ cause might be considered functional overload of involved tissues [4].

Therapy of temporomandibular disorders (TMD) involves a complex healing process mainly due to the multifactorial etiology of the disease. The etiological factors include genetic conditions, factors located inside the oral cavity and environmental determinants, with particular emphasis on psychoemotional factors that were proven to have a decisive influence on the elevated muscle activity in the masticatory organ [5–7].

The key in the treatment of TMDis to provide the patient with specialist dental care in order to restore intra-oral homeostasis, counselling in order to diagnose and treat psycho-emotional disorders and physiotherapeutic care, orofacial pain treatment focused on pain elimination, as well as the improvement or restoration of the regular movement pattern within TMJ and adjacent tissues [8].

Due to the type of the therapeutic method used for TMD, procedures applied in the process can be divided into: dental (e.g. occlusal appliances, long-term irreversible prosthetic treatment, orthodontic alignment) [9, 10] local or systemic pharmacological approach (e.g. botulinum toxin injections/NSAIDS etc.) [11], physiotherapeutic (manual therapy, physical therapy, needle therapy) [12, 13] and psychological (e.g. autogenic training) [14]. It should be emphasized that an increasing number of clinicians use interdisciplinary treatment by combining these methods [15].

In medical literature, the role of physiotherapy for the recovery of patients with TMD is emphasized [16]. Modern physiotherapy has a wide range of therapeutic techniques. In the traditional division, they are classified as: physical therapy, kinesiotherapy and massage. Since the 1990s, there have been changes in physiotherapy, which were associated with the opening to new methods of working with the patient, including manual therapy - MT.

According to the current definition provided by IFOMPT (International Federation of Orthopedic Manipulative Physical Therapists), MT should be understood as: ‘a specialized area of physiotherapy, devoted to the management of neuromuscular-skeletal diseases, based on clinical conclusions and the use of highly specialized methods of treatment, including manual techniques and therapeutic exercises’ [17]. MT has been divided into two branches of development- ‘soft’, devoted to the treatment of soft tissues including post-isometric muscle relaxation (PIR), deep tissue massage, myofascial relaxation, transverse massage, positional relaxation, fascial therapy; and ‘joint’ (‘hard’) which focuses on the hard tissues of the body. It is based on the performance of manipulation, i.e. a technique of rapidly exceeding the physiological range of motion in a joint, using high speed, but with a small amplitude [18].

Articular techniques dominated in MT until recently. Currently, it is believed that this way of management is only acceptable in the acute phase, therefore not used for chronic pain [19]. Taking into account the pain and increased muscle tension and the fascia integrated with them, it seems the right view of the use of soft tissue therapy in patients with TMD [20, 21].

Modern physical therapy makes it possible to apply two physical factors simultaneously [22]. Hence, various forms of physical therapy are often applied in addition to manual techniques. This may make the fight against pain more effective. In dentistry, for therapeutic purposes, forms of physical therapy are increasingly used, such as: slowly changing magnetic field, laser, electric currents, variety of light or associated factors [23, 24]. They create additional possibilities of therapeutic applications, which extend the existing range of therapeutic effects in dental issues.

The effectiveness of TMD treatment largely depends on the proper problem assessment and initial diagnosis. Orofacial pain specialist integrates patients’ medical history, physical examination and imaging prior to treatment plan implementation.

In order to standardize the diagnostic criteria for TMD, a standardized tool, the Diagnostic Criteria for Temporomandibular Joint Disorders (DC-TMD), was established in 2014. Based on the patient’s signs and symptoms, DC/TMD defines two axes: (i) Axis I, dividing TMD into muscular (Group I) and atherogenic TMD (Groups II and III); Axis II, assessing behavioral and psychological status [25].

Skeletal muscle palpation in standard diagnostic methods allows only for subjective, operator-biased assessment of elevated muscle tension and tenderness to palpation. Currently, thanks to the development of medical physics and biomedical engineering, it is possible to use additional functional diagnostic methods into our advantage, which is considered by many a valuable addition to clinical judgment process and proper patient management. One of the quantitative tools that significantly complement the examination of the masticatory muscle function is surface electromyography - sEMG [26], less invasive version of older examination, known as needle electromyography [27].

The sEMG assessment of masticatory muscle function in people with TMD forms the basis for diagnosing the disease, monitoring its progression and measuring the effectiveness of treatment. Numerous studies have shown that, in patients with TMD, changes in masticatory muscle EMG activity are either due to the disease itself or to a compensatory mechanism related to symptoms [28–30]. It has been shown that individuals with pain-related TMD can alter masticatory muscle recruitment through sensorimotor interactions, and associated pain can modify action potential generation and possibly myoelectric activity [31]. Studies evaluating the usefulness of sEMG in the diagnosis and assessment of physiotherapy effects in patients with TMD pain and limited TMJ mobility suggest that exercise EMG (MVC) is a method that is worth including in the diagnosis and therapy process (PC1 99%) [21].

In most up-to-date literature, there are just a few randomized controlled trials (RCTs) comparing the effectiveness of treatment with MT and physical therapy in TMD pain. This prompted the authors to investigate the topic of the analgesic application of manual therapy, magnetostimulation and synergism of physical factors in patients with TMD. Due to the wide variety of symptoms experienced by patients with TMJ disorders, the studies focused on three commonly reported conditions. These include: chronic pain in the masseter muscles (over 3 months), limited mobility of the mandible and a feeling of increased tension in the masseter muscle.

We hypothesized that physical and manual therapeutic factors alter the bioelectrical activity of the masseter muscle, which could contribute to the improvement of TMJ mobility. Reducing muscle tension reduces the intensity of the perceived pain.

The aim of the study is to test the effectiveness of the manual factors used.

and physical exercises in the elimination of pain and increased muscle tension in patients.

with TMD. Additionally, the assessment of the influence of the applied forms of therapy on the TMJ range of motion (ROM).

## Methods

### Trial design

**Fig. 1:**
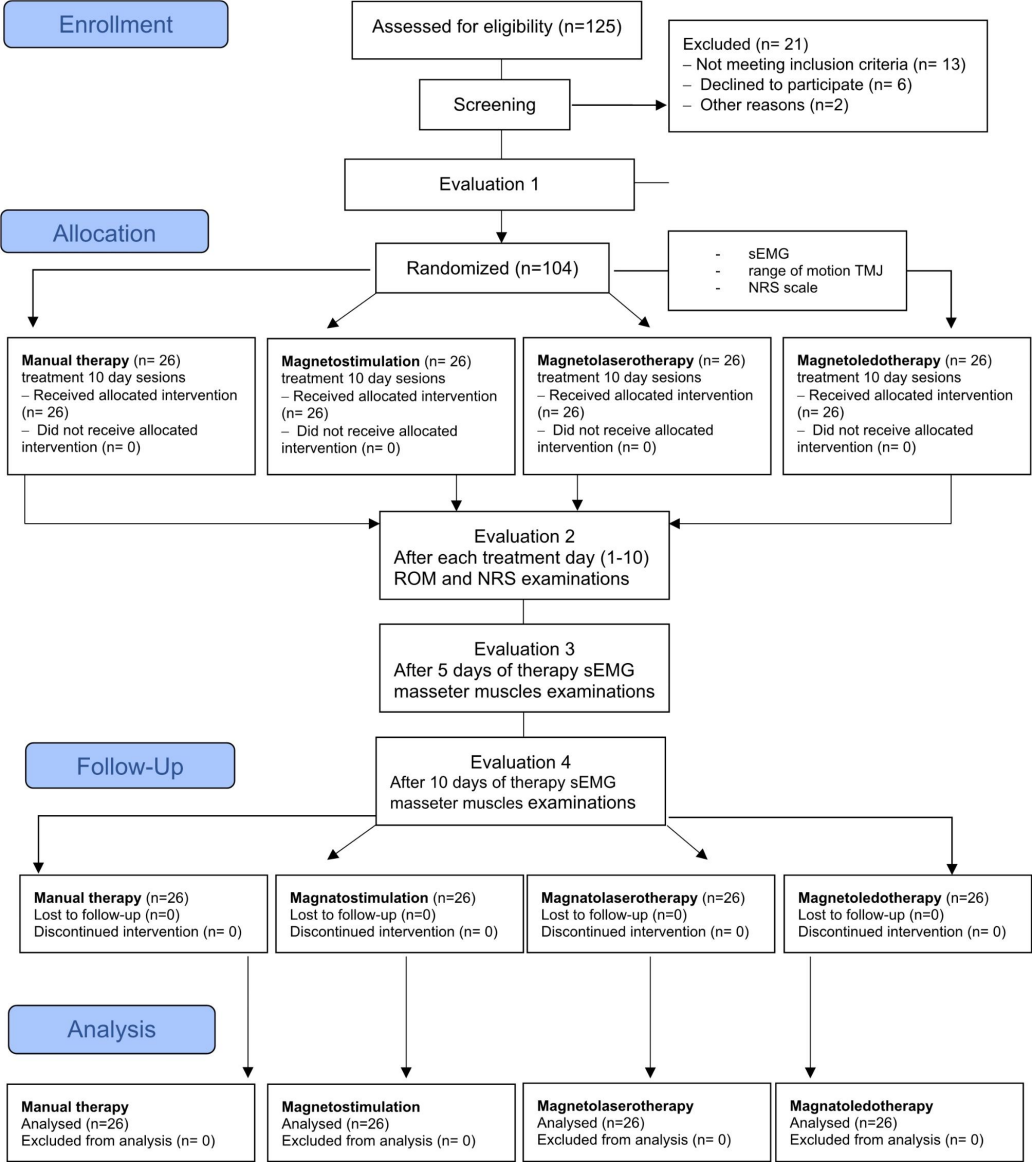
CONSORT flowchart of the participants’ progress through the trial phases [32]

This parallel randomized clinical trial (RCT), four-arm with equal allocation ratio (1:1), controlled study followed the Standards of Study Reporting (CONSORT) [32]. The study was conducted on patients from the University Dental Clinic at the Pomeranian Medical University in Szczecin (Poland). Patients were divided into four groups in which physiotherapy was carried out for 10 days (excluding Saturdays and Sundays). All interventions performed in all study groups were performed at no cost, under the same conditions and by the same physiotherapist. The study protocol is presented in Fig. 1.

The research project was approved by the Bioethics Committee of the Pomeranian Medical University in Szczecin (no. KB – 0012/102/13). Trial was registered (date 19/08/2021) at www.ClinicalTrials.gov database (NCT05021874).

### Participants

The study included 104 women aged 20 to 45 years who, based on DC/TMD criteria, were diagnosed with myofascial pain with mouth opening restriction, for longer than 3 months (Ib). Patients were randomly assigned (simple randomization) to the experimental four therapeutic group i.e.: magnetostimulation (MS, n = 26), magnetoledotherapy (MLE, n = 26), magnetolaserotherapy (MLA, n = 26), manual therapy (MT, n = 26). None of the respondents refused to participate in the study.

Study exclusion criteria were as follows: inflammation in the oral cavity that emerged as myospasm or preventive muscle contraction, earlier splint therapy, pharmacotherapy (e.g., oral contraception, hormone replacement therapy, and antidepressants), systemic diseases (e.g., rheumatic and metabolic diseases), mental illness, lack of mandible orthopedic stability, masticatory organ or whiplash injury, pregnancy, patients undergoing orthodontic treatment, other types of inflammation in the oral cavity (e.g., pulp inflammation or impacted molars), and fibromyalgia;dermatology disease, contraindications to the use of physical treatments in the field of magneto-stimulation, light therapy or manual therapy.

All women underwent an intra-oral and extra-oral dental examination performed by an Orofacial Pain trained dentist. The aim was to exclude odontogenic, periodontal and intracapsular origins of TMD pain.

The control group (G2) consisted of 104 healthy women, aged 20 to 45, without claimed TMD and pain disorders (intra-oral and extra-oral dental examination).

The determination of whether the patient met the inclusion criteria on the basis of the subject and physical examination was made by a dentist. Afterwards, another dentist participated to randomize the patients.

The patients qualified for the study underwent instrumental diagnostics (sEMG of the masseter muscles at rest and exercise, linear measurement of the range of mandibular mobility) and the level of pain intensity was assessed on the NRS numerical scale. Then, in G1, ROM and NRS measurements were performed each time after treatment, and sEMG after 5 and 10 days of treatment.

The study was conducted from January 2022 to February 2023 on patients of the University Dental Clinic of the Pomeranian Medical University in Szczecin.

The activities undertaken by the investigators during the trial are presented in Table 1.

**Table 1:** Activities of investigators during the trial

Day	0	1	2	3	4	5	6	7	8	9	10
Screening and inclusion	+										
Measure sEMG	+					+					+
Measure NRS	+	+	+	+	+	+	+	+	+	+	+
Measure ROM	+	+	+	+	+	+	+	+	+	+	+
Therapy		+	+	+	+	+	+	+	+	+	+

Legend: 0 - preliminary examination, 10 – the last day of therapy and final examination

### Interventions

In group G1, masseter muscles physiotherapy was performed for 10 days (excluding Saturdays and Sundays). The division into groups was as follows:

- *MS (magnetostimulation)*.

A pulsating, heterogeneous magnetic field generated by the apparatus using an elliptical applicator with a beam width of approx. 5 cm. In this subgroup, a therapeutic program was prescribed of increasing intensity from “3” to “7”, increasing by one level, every other day. The elliptical applicator remained stationary in the same place during the treatment [33].

*b) MLE* (magnetoledotherapy).

Synergic action of a slow-changing magnetic field and LEDs with a wavelength of 860 nm. An elliptical magnetic - light (IR) applicator with a diameter of 5 cm containing 47 infrared diodes was used. The method of applying the applicator, the type of program, its application and the duration of the procedure were the same as in the MS subgroup [34].

*c) MLA* (magnetolaserotherapy).

Synergic operation of a slowly changing magnetic field and a low-energy IR laser, with a wavelength of 808 nm, maximum power of 300 mW and frequency of 181.8 Hz. During the procedure, it was important to observe the principle of perpendicular incidence of the radiation beam on the tissue and the use of protective glasses by the patient and the therapist. The patients were dosed with laser radiation with an increasing intensity from 3.0 J/cm^2^ to 5.0 J/cm^2^ (with an interval of 2 days, the intensity was increased by 0.5 J/cm^2^). The total dose of laser radiation administered to each patient was 40 J/cm^2^ [34].

d) *MT* (manual therapy).

Extraoral and intraoral therapy within the masseter muscles. The following techniques of work on soft tissues were used during the therapy: compressive mobilization of myofascial trigger points (mTRPs) within the masseter muscle, mobilization of the cell and pain spheres of the facial subcutaneous tissue with the Kibler fold, functional massage of the masseter muscle (massage of the muscle and other surrounding soft tissues in conjunction with the movement of abduction and adduction of the mandible), post-isometric relaxation of the masseter muscle [35–37].

Physical procedures were performed using the Viofor JPS Clinic (Med & Life, Komorow, Poland) (in the case of MA and MLE) and the Viofor JPS Clinic model connected via a link with the Viofor Laser JPS apparatus (Med & Life, Komorow, Poland) (in the case of MLA). In each treatment group, the duration of the procedure within one masseter muscle was 12 min.

### Outcomes

#### Primary outcome measures

*Pain severity scale*.

In women from G1, pain was assessed on the NRS scale (pain severity scale from 0 to 10, with zero meaning “no pain” and 10 meaning “the worst pain imaginable) each time after therapy and the range of mandibular mobility was assessed. After treatment days 5 and 10, a follow-up sEMG study was performed at rest and exercise.

*Range of motion temporomandibular joint*.

- Measurement of mouth opening (MMO): the patient was in a sitting position during the measurement. Millimeter ruler was placed at the incisaledge of the maxillary central incisor thatis the most vertically oriented and mea-sured vertically to the labioincisal edge ofthe opposing mandibular incisor. Theamount of vertical incisor overlap wasadded to each of these measurements todetermine the actual amount of opening [38].
- Measurement of lateral movements: the patient was in a sitting position during the measurement. Measurement of lateral movements– subject opened slightly (physiologicalrest position) and moved the mandible asfar as possible toward the right or left. Itwas measured by means of the millimeterruler from the labioincisal embrasure be-tween the maxillary central incisors tothe labioincisal embrasure of the mandib-ular incisors [38].

*sEMG test of masseter muscle (MAS)*.

All patients were assessed for MAS muscles sEMG, during the preliminary examination, after the 5th and 10th day of treatment.

SEMG recordings from the masseter muscles were performed with a two-channel NeuroTrac MyoPlus 2 device with NeuroTrac software (Verity Medical Ltd., Tagoat, Ireland). Clinical Mode EMG was used during the study. To obtain precise sEMG measurements, a band-stop filter was used, which guarantees that the frequencies of 50 and 60 Hz (mains) will not interfere with the recording of muscle activity (measured in microvolts). Specialized filtering allows sEMG to be measured with a precision of 0.1 µV.

In order to avoid magnetic interference, when collecting sEMG measurements, the device was not placed in the vicinity of cell phones (< 4 m) or other sources that might interfere with the results. Two unipolar electrodes were used for the test, which were attached at a distance of 10 mm from each other. The main principle used in all subjects was to position the electrodes over the center of muscle belly, parallel to the path of its fibers (the lower electrode was placed approx. 5 mm above the mandibular angle and the upper electrode 10 mm above it); precise placement of the electrodes was preceded by careful palpation of each muscle. The masseter muscles bioelectrical signals were acquired in an upright sitting position, with the head in a natural, postural position, hands resting on the knees and feet resting on the ground. Before the electrodes were applied, the skin was cleaned with rubbing alcohol disinfectant, following electrode manufacturers’ manual and guidelines of Seniam project (www.seniam.org). The neutral electrode was located on the cervical section - C7 styloid process, which is usually devoid of vastly active muscle fibers.

- Examination of the electrical activity of the masseter muscle at rest (Rest Test): the test was performed on relaxed and relaxed patients. The dental arches remained slightly open during the examination. The patients were instructed not to swallow saliva during the examination and to place their tongue in a resting position [39].
- Study of the bioelectrical activity of the masseter muscle during maximal voluntary contraction (MVC): sEMG signal was recorded in a sitting position, while clenching the teeth, using the greatest possible force, within 5 s. The computer program with which the device cooperated registers the minimum and maximum values and calculates the average values of electric potentials [39].

The sEMG values obtained were normalized as the ratio of RLX to MVC.

Activity normalized to MVC [%] = RLX [µV]/ MVC [µV] x 100% [40].

### Sample size

Sample size was determined for Anova repated measurements model with within-between interaction at effect size 0.25, alpfa equal to 0.05 and power equal 0.80 total size requred was equal to 24 [41].

### Randomization and blinding

Patients were randomly assigned (simple randomization) to the study group using the closed envelope method. In order to conceal the allocation (1:1), consecutively numbered, opaque, sealed envelopes were used, and care was taken to ensure that they were undamaged and not see-through when held against a light source. Randomisation was carried out by an investigator who was not involved in patient eligibility, intervention delivery or data collection.

Outcome assessors were blind to group allocation, and were not involved in providing the interventions (single-blind). The statisticians conducting the statistical analyses were blind to group allocation until after the analyses were completed. It was not necessary to unblind any of the participants at any time during the study.

### Statistical analysis

The data were presented as a median with a quartile range due to significant deviations from the normal distribution assessed using the graphical method with the analysis of the histogram and the Q-Q plot. Due to significant deviations from the normal distribution, the dependence of the studied variables on time and between groups was performed based on the nparLD test, which is a non-parametric equivalent of the analysis of variance for repeated measurements. The Kruskall Wallis test with the post-Hoc multiple comparison test was used to compare the baseline values of the parameters studied for the control group. Comparisons between the control group and the total study group were made using the U-Mann Whitney test. The values of p < 0.05 were considered significant. The analysis was performed in the R language in the RStudio software using the NparLD, ggplot2, ggpubr libraries [42].

## Results

### Participant flow

The flow of patients through the study, according to CONSORT criteria, is reported below (Fig. 1). One hundred and twenty five subjects were screened over a period of 5 months, and twenty-one were excluded because they did not meet the inclusion criteria and/or met one or more exclusion criteria.

Figure 1. CONSORT flowchart of the participants’ progress through the trial phases [27].

### Recruitment

Participants meeting the inclusion criteria were recruited between August 2021 and January 2022. Patients attended clinic visits at the time of randomization (baseline) and at 5-day and 10-day intervals from the baseline day.

### Baseline data

The results of the preliminary study in the test and control groups are presented in Table 2.

**Table 2:** Statistical analysis of the age structure, sEMG values, TMJ range of motion, and pain intensity in the G1 and G2 groups at baseline

Variable		G1 (study group)				G2 (control group)				p
		Me	Q1	Q3	95%CI	Me	Q1	Q3	95%CI	
Age		29.50	24.50	38.50		29.00	25.00	39.00		0.51
MMO [mm]		36.50	35.00	38.00	36.11–36.69	45.00	44.00	46.00	44.63–45.06	0.00
LMR [mm]		6.00	6.00	7.00	6.00-6.33	10.00	9.00	10.00	9.43–9.89	0.00
LML [mm]		6.00	5.00	6.00	5.78–6.13	10.00	9.00	11.00	9.47–10.03	0.00
NRS scale		6.00	6.00	7.00	6.02–6.38	0.00	0.00	0.00	0.0–0.0	0.00
sEMG (RLX Test) [µV]	right	27.70	19.30	36.50	27.59–33.07	1.85	1.30	2.35	1.67–1.92	0.00
	left	43.40	31.50	55.75	41.98–49.27	1.60	1.30	2.25	1.59–1.84	0.00
sEMG (MVC) [µV]	right	284.50	218.50	374.50	275.04-314.95	66.90	58.90	76.65	65.59–69.09	0.00
	left	294	232	369.50	286.21–321.80	66.90	60.15	75.40	65.39–68.88	0.00
sEMG (%MVC)	right	41.3	9.82	6.66	0.691–1.37	5.23	2.66	1.85	0.09–0.18	0.00
	left	52.4	14.3	10.1	0.93–1.84	5.43	2.44	1.82	0.10–0.20	0.00

Legend: MMO –Maximal Mouth Opening, LMR – right lateral movement, LML - left lateral movement, NRS- Numeric Rating Scale, RLX test – Rest test, MVC – Maximum Voluntary Contractions

**Table 3:** Confidence interval (CI 95%) after the 10th treatment in the G1 group

95%CL					
Therapy group		MS	MLE	MLA	MT
MMO [mm]		36.06–37.39	36.81–38.03	36.68–38.08	42.58–43.34
LMR [mm]		5.95–6.58	6.46–7.31	6.29–6.86	8,09 − 8,91
LML [mm]		5.80–6.58	6.18–7.05	5.76–6.24	8.14–8.86
NRS scale		4.55–5.29	2.67–3.32	2.66–3.11	0.03–0.18
sEMG (RLX Test) [µV]	Right	23.40-37.48	20.42–29.22	18.35–30.22	3.57–5.17
	Left	35.77–53.43	36.49–52.49	35.34–49.10	3.53–4.95
sEMG (MVC) [µV]	Right	218.61-322.35	216.06-296.55	246.55-343.53	69.62–78.91
	Left	291.29-377.17	238.69-313.31	261.66-323.96	68.91–77.87

Legend: MMO –Maximal Mouth Opening, LMR – right lateral movement, LML - left lateral movement, NRS- Numeric Rating Scale, RLX test – Rest test, MVC – Maximum Voluntary Contractions, MT- manual therapy, MS – magnetostimulation, MLA - magnetolaserotherapy, MLE – magnetoledotherapy

As shown in Table 2, there was no statistical difference in the age structure between the groups (p = 0.51). On the other hand, there was a statistical difference between the groups during the initial sEMG examination of the masseter muscle (p = 0.00), in the mobility of the TMJ (p = 0.00) and the level of pain intensity (p = 0.00) (Table 2).

### Numbers analysed

The primary analysis was intention-to-treat and included all patients who were randomly assigned. Patients in groups G1(n = 104) and G2 (n = 104) were analyzed according to the protocol.

### Outcomes and estimation

#### Primary outcome

**Fig. 2:**
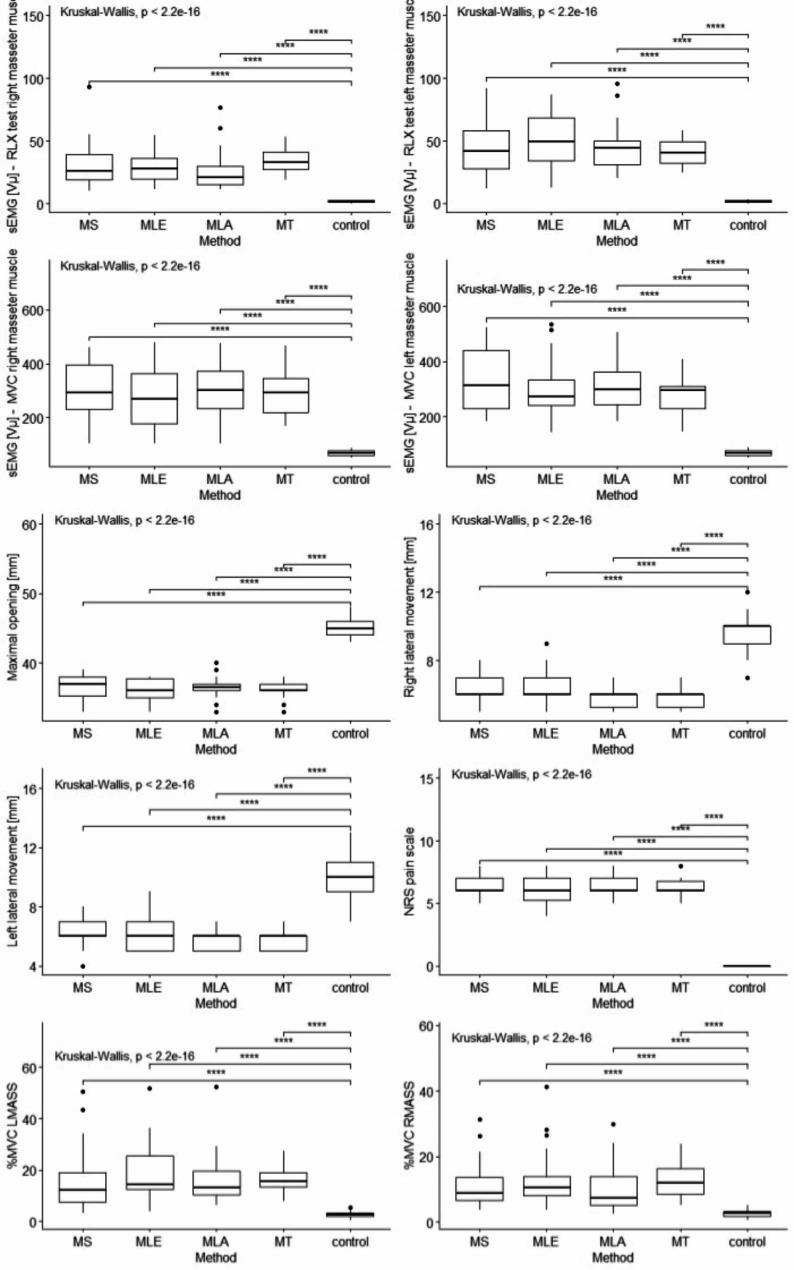
Statistical analysis of the sEMG distribution, mandibular mobility range and pain intensity in the treatment groups and the control group at baseline Legend: MVC- Maximal Voluntary Contractions; RLX test – Rest test; RMASS – right masseter muscle, LMASS – left masseter muscle, MT- manual therapy, MS – magnetostimulation, MLA – magnetolaserotherapy, MLE – magnetoledotherapy, control – control group symnum.args a list of arguments to pass to the function symnum for symbolic number coding of p-values. For example, symnum.args <- list(cutpoints = c(0, 0.0001, 0.001, 0.01, 0.05, 1), symbols = c(“****”, “***”, “**”, “*”, “ns”)).In other words, we use the following convention for symbols indicating statistical significance: ns: p > 0.05*: p < = 0.05; **: p < = 0.01; ***: p < = 0.001; ****: p < = 0.0001

Fig. 2 presents a comparative analysis of variables (sEMG, MMO, LMR, LML, NRS) in therapeutic subgroups and in the control group.

Significantly higher results were observed in all sEMG measurements in each therapeutic subgroup compared to the control group (G2). The range of mandibular abduction and lateral mandibular movements in the control group were significantly higher than in the study groups. Similarly, in the case of the pain intensity scale, where in the control group all respondents scored ‘0’ on the NRS scale, while among the respondents from the study group, the pain intensity values were significantly higher in each subgroup (Fig. 2).

#### Secondary outcome

**Fig. 3:**
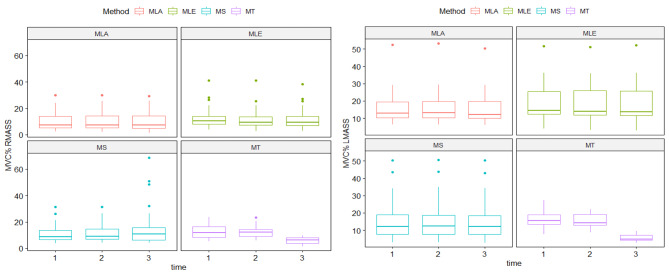
Changes of sEMG median during study in each therapeutic group p_group, p_time p_time*group p < 0.001 Legend: MVC% - Maximal Voluntary Contractions, RMASS – right masseter muscle, LMASS – left masseter muscle, MT- manual therapy, MS – magnetostimulation, MLA – magnetolaserotherapy, MLE – magnetoledotherapy

Figure 3 below shows the effect of the applied physiotherapeutic treatments in all four therapeutic subgroups on sEMG (MVC%) during the initial study (1), after 5 days of therapy (2), and after the 10th treatment day (3).

As presented in Fig. 3, the greatest decrease in sEMG of the masseter muscle occurred in the subgroup in which the MT procedures were applied, p < 0.000 in post hoc test. There was no clinically significant difference in other subgroups (Fig. 3).

**Fig. 4:**
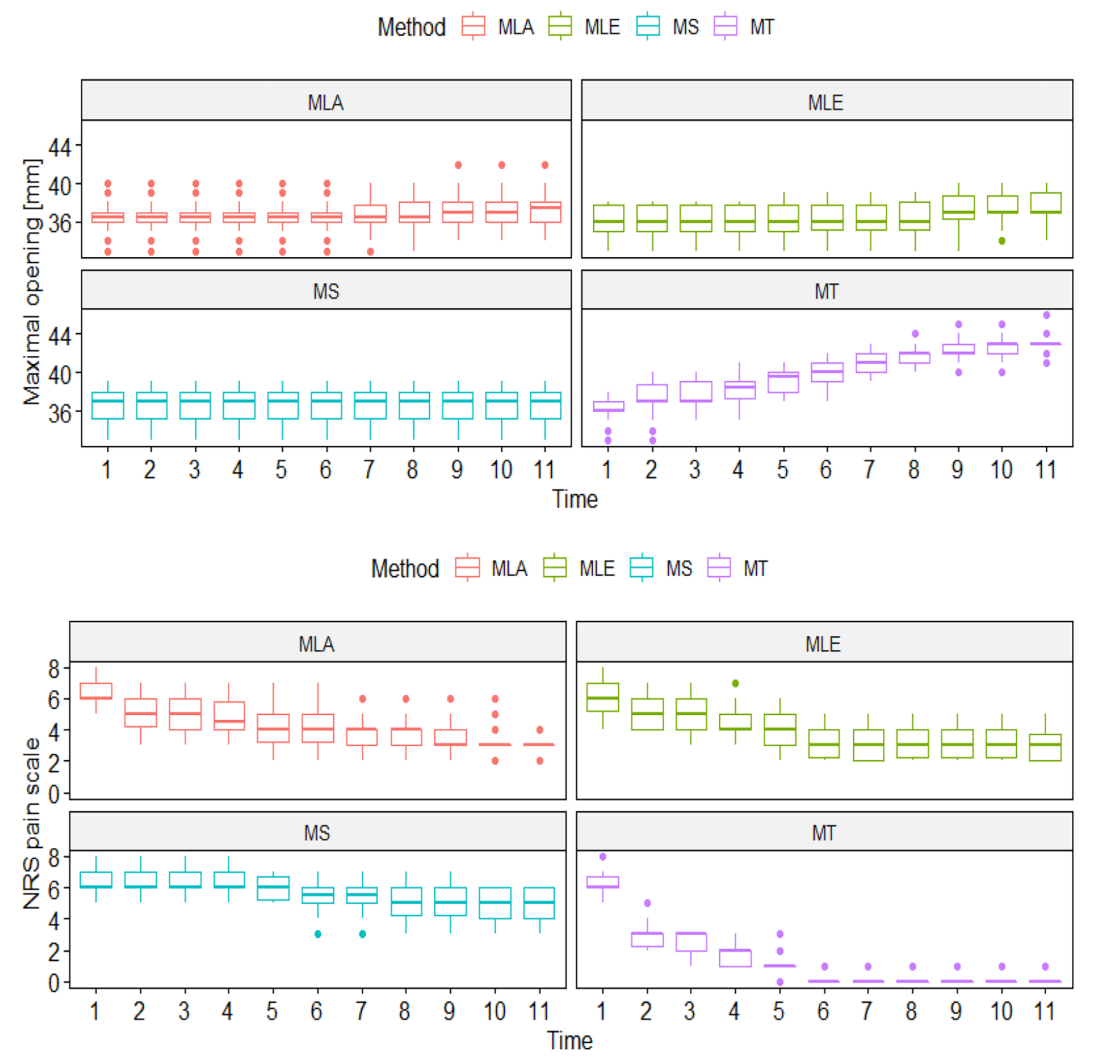
Changes of movement range of the abduction of the mandible and pain intensity in the G1 group during study Legend: MT- manual therapy, MS – magnetostimulation, MLA – magnetolaserotherapy, MLE – magnetoledotherapy

Figure 4 shows the 10-day analysis (1 - initial examination, 11 - tenth day of therapy), the range of mandibular mobility in four therapeutic subgroups and the intensity of pain on the NRS scale.

There was a clear increase in abduction of the mandible in the test group undergoing MT, with minimal changes in the MLA and MLE groups and no changes in the MS group. In the case of pain measurements, the greatest improvement was observed among MT respondents, where pain sensations did not exceed 1 on the NRS scale from day 6. In the other groups, the decrease in pain level over the course of the study was small (Fig. 4).

**Fig. 5:**
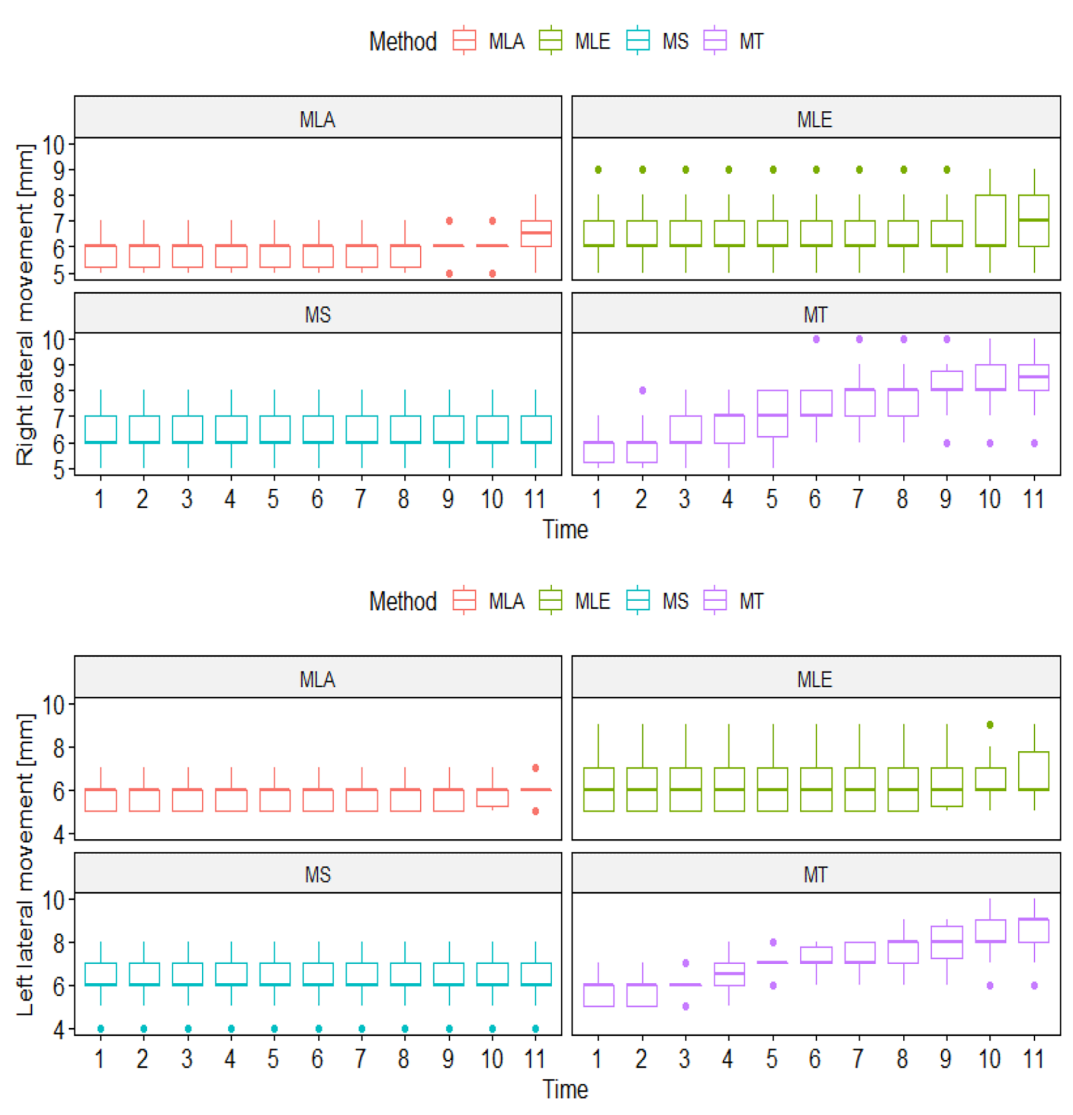
Changes of lateral movement in the G1 group during study Legend: MT- manual therapy, MS – magnetostimulation, MLA – magnetolaserotherapy, MLE – magnetoledotherapy

Figure 5 shows a 10-day (1-initial examination, 11 - tenth day of therapy) analysis of the range of lateral mandibular movements in four therapeutic subgroups.

There was a clear increase in both right and left lateral mobility in the MT group. There were no differences in the course of the study in the MS group, and slight increases in the MLA and MLE groups. The significant effect of the interaction of time and method indicates the highest effectiveness of manual therapy among the methods tested (Fig. 5).

Table 3 presents the confidence interval obtained after the 10th physiotherapy treatment in the study group (G1).

### Ancillary analyses

Table 4 presents changes in the test values of the test parameters over the course of the study, obtained by subtracting the result after the last one treatment from the result before the first procedure.

**Table 4:** Change in the values of the test parameters over the course of the study, obtained by subtracting the result after the 10th treatment from the result before the first procedure

Variable	MLA			MLE			MS			MT		
	Me	Q1	Q3	Me	Q1	Q3	Me	Q1	Q3	Me	Q1	Q3
d_lateral right	1	0	1	0	0	0,75	0	0	0	3	2	3
d_NRS	-3	-4	-3	-3	-4	-3	-1	-2	-1	-6	-6	-6
d_mouth open	1	0	1	1	1	2	0	0	0	7	6	8
dsEMG (RLX Test) [µV] left	-2,1	-2,98	-1,3	-5,7	-7,48	-4,82	-1	-1,3	-0,525	-36,7	-43,7	-29
dsEMG (RLX Test) [µV] right	-1,8	-3,1	-1,3	-5,1	-5,6	-4,4	-0,65	-1,18	-0,425	-28,6	-33,9	-23,8
dsEMG (MVC) [µV] left	-10	-11,8	-9	-18	-27	-14	-5	-6	-3	-208	-238	-141
dsEMG (MVC) [µV] right	-11	-13	-10	-17,5	-25,8	-12,2	-5	-6,75	-4	-215	-271	-145
%MVC sEMG LMASS	-0,15	-0,548	0,02	-0,845	-1,33	-0,26	-0,12	-0,29	0,015	-9,76	-13,1	-6,6
%MVC sEMG RMASS	-0,22	-0,58	0,02	-1,14	-1,57	-0,8	-0,03	-0,19	0,173	-6,4	-10,2	-3,33

Legend: MT- manual therapy, MS – magnetostimulation, MLA – magnetolaserotherapy, MLE – magnetoledotherapy, Me- median, Q1- first quartile, Q3 – third quartile, d – delta, RLX test – Rest test, %MVC - Maximal Voluntary Contractions, RMASS – right masseter muscle, LMASS – left masseter muscle

As presented in Table 4 MT procedure led to a decrease of 6 points on the NRS scale, where with physical treatments the decrease was clinically much smaller. TM procedures led to the greatest progression in terms of increasing MO movement and contributed to a decrease in the bioelectrical activity of the masseter muscles (Table 4).

### Possible harms

No patient reported adverse effects throughout the study. Thus, zero adverse events were reported during the conduct of the study.

## Discussion

A limitation in the conducted studies was the short duration of therapy which women underwent and the small number of therapeutic subgroups. Additionally, no assessment was made on how long the therapeutic effect obtained during the applied treatments was maintained after the end of the treatment cycle. Therefore, the authors see the need to continue the conducted research, focusing on the above limitations.

Non-invasive therapeutic methods play an important role in the treatment of TMD. In TMD, where the pain triggering factor is an increased muscle tone, the first treatment should be implemented to eliminate these ailments. At the initial stage of treatment, it is worth using physiotherapeutic methods. Thanks to them, may obtain analgesic and myorelaxing effect. The next step should be to coordinate appropriate dental treatment, i.e. splint therapy [39]. While rehabilitation in the form of splints has an established position in modern dental treatment, the use of physiotherapeutic treatment in patients with TMD still requires numerous clinical trials to determine their therapeutic effectiveness.

In this research, the authors focused on the assessment of the effectiveness of the manual therapy and physical therapy procedures, and their clinical effectiveness was assessed on the basis of the analysis of non-invasive sEMG of the masseter muscles, the assessment of pain intensity on the NRS scale and the assessment of mandibular mobility.

According to the preliminary examination, patients with pain and limited mobility of the mandible at baseline had statistically significantly higher sEMG values of the masseter muscle compared to the control group (p < 0.00; sEMG RLX right IC95% 23.40-37.48 and left IC95% 35.77–53.43; sEMG MVC right IC95% 218.61-322.35 and left IC95% 291.29-377.17) before the start of physiotherapy. On the other hand, all assessed ranges of mandibular mobility (opening, lateral movements to the right and left) were significantly smaller in the study group (p < 0.00). When characterizing pain intensity in the study group, its mean value was 6 NRS (moderate pain). As shown by other authors, increased muscle tension affects the range of mobility of the temporomandibular joints and contributes to the occurrence of pain [43].

According to the studies conducted by the authors, MT turned out to be the most effective therapeutic method in terms of analgesic and myorelaxation. In patients who underwent physical therapy procedures, no clinically significant difference in the assessed parameters was found. This data may indicate the superiority of manual methods over physical methods in patients with TMD disorders. Hence, in the opinion of the authors, physical methods should only complement the therapy in manual work with TMD patients. Particularly noteworthy is the fact that after the 6th MT treatment the patients had complete pain relief (0 NRS) or the pain remained at the level of 1 on the NRS scale. For comparison, in patients who underwent physical procedures, the intensity of pain on the 6th day of therapy was: MS (NRS 7), MLA (NRS 6), and MLE (NRS 5), respectively. According to studies by Urbański et al., the use of MT treatments (PIR or MR- myofascial release treatment) in patients with TMD contributes to a decrease in the bioelectric activity of the masticatory muscles and a decrease in the intensity of pain in the VAS scale [44]. Manzotti et al. assessed the therapeutic effectiveness of manipulative osteopathic treatment on the activity of the muscles of the stomatognathic apparatus, according to their research, the above form of therapy significantly influences the bioelectric activity of the masseter muscles compared to the placebo group [45]. Rodrigues D et al., when assessing the impact of tens treatments on the bioelectric activity of masticatory muscles and pain, observed that the application of tens effectively reduces pain, but does not act homogeneously on the characteristics of electrical activity of the assessed muscles [46]. Similarly to the above, the effectiveness of the analgesic effect of physical therapy was described by Del Vecchio et al., indicating the effectiveness of home treatment of pain associated with TMD using the method of low energy laser therapy (LLLT) [47]. Chellappa et al. investigated that tens and LLLT therapy improves the range of TMJ movement and relieves pain in both therapies with the predominant effectiveness of LLLT therapy [48]. Sancakli et al. assessed the effectiveness of the use of a low-level laser (LLL) on the points of greatest pain in patients with chronic pain in the masticatory muscles. According to their research, as a result of the applied physical therapy, there was a statistically significant reduction in the value of PPT of muscles, the number of muscles without pain on palpation increased significantly, and the range of mandibular movements improved. In contrast, the placebo group showed no statistically significant difference in any of the measured values [49].

Analyzing the above data and our own results, it can be stated with great caution that treatments in the field of physical therapy reduce the intensity of the perceived pain, and thus improve the range of mandibular mobility, but their effect does not affect the bioelectrical activity of the muscle. On the other hand, the effect of MT, due to its deeper impact on soft tissues, in addition to improving the TMJ function and analgesic effect, leads to changes in the sEMG parameters.

The lack of systematic knowledge about the effectiveness of MT in people with TMD prompted Calixtre et al. [50] to conduct a systematic review of the scientific evidence regarding the isolated effect of MT on the improvement of maximum mouth opening and pain in patients with TMD symptoms. According to it, there is very different evidence that MT (depending on the technique) relieves pain, reduces the pressure threshold of the tissue, and affects the range of motion of TMJ in people with TMD symptoms. According to the analysis, myofascial relaxation and massage techniques applied to the masticatory muscles are more effective than control, but as effective as botulinum toxin injections. Techniques for manipulating or mobilizing the upper cervical spine are more effective than control, while chest manipulations are not [50]. The difficulty in comparing the available research results with the results of the authors of the study is caused by the lack of standardized assessments and protocols for physiotherapeutic treatment, and this filling of the gap would significantly strengthen the clinical significance of the conducted research.

A very valuable meta-analysis was performed by Zangh et al. in which they compared the impact of therapeutic exercises and bite-bar therapy on pain and mobility in people with painful TMJ disorders. The conducted analysis did not show high-quality evidence to distinguish clinical efficacy between occlusal splint therapy and exercise therapy in patients with painful TMD and limited mandibular mobility. Hence, it seems necessary to implement more randomized, controlled trials comparing the effects of TMJ exercise and treatment of occlusal splints [51].

When analyzing the TMJ mobility parameter in the conducted studies, also in this case the most effective form of therapy turned out to be the MT. The statistical analysis shows that 10 MT treatments lead to a significant improvement in TMD function, which is confirmed by the obtained parameters of MMO (MT = 42.96 mm; 95%CI 42.58–43.34) and lateral movements of the mandible (LMR = 8.50 mm, 95%CI 8.09-8.91and LML = 8.63 mm, 95% CI 8.14–8.86). In patients undergoing physical procedures after 10 days of treatment, no clinically significant therapeutic effects were observed, i.e. MS (MMO = 36.73, 95%CI 36.06–37.39;

LMR = 6.27 mm, 95%CI 5.95–6.58 and LRL = 6.19.mm, 95%CI 5.80–6.58) MLE (MMO = 37.42 mm, 95%CI 36.81–38.03; LMR = 6.88 mm, 95%CI 6.46-7.31and LRL = 6.62 mm, 95%CI 6.18–7.05) MLA (MMO = 37.38 mm, 95%CI 36.68–38.08; LMR = 6.58 mm, 95%CI 6.29–6.86 and LRL = 6.00 mm, 95%CI 5.76–6.24). Similar results were obtained by Tuncer et al., who showed that a more effective therapeutic effect in the treatment of TMJ dysfunction is achieved through MT, combined with home physical therapy than through monotherapy (physical therapy) [52].

From the clinical point of view, the research conducted by the authors shows the legitimacy of implementing manual therapy treatments into everyday physiotherapeutic practice in patients with TMD. As can be seen from the analysis of the change in the values of the parameters studied during the study, obtained by subtracting the result after the 10th treatment from the result before the first procedure, from a clinical point of view, there was a significant saucer of pain intensity (-6NRS) and bioelectrical function of the masseter muscle (%MVC P=-6.4/L=-9.76) and an increase in the extent of mandibular visitation (7 mm) in the TM group. No clinically significant differences in the parameters studied were observed in any group where physical treatments were applied.

Comparing the duration of therapy in our study with those of other authors, we can observe that there are large differences in the literature on this variable. In addition, there is often no information as to whether the therapy was conducted at the weekend. However, we saw two similar studies on physiotherapy in TMD patients that were similar to ours in terms of the duration of treatments. In the study by Urbanski et al. [44], this time was, as in our study, 10 days (excluding Sundays); however, in our study the days excluded from therapy were both Saturday and Sunday. This is due to the most common physiotherapy practice where treatment services are not provided on public holidays (e.g. weekends). We can observe a similar treatment approach in the study by Kubala et al. [34]. In this study, therapy (magnetostimulation or magnetoledotherapy) in patients with TMD was given for 3 weeks, with daily treatments excluding weekends (15 treatments in total).

Therefore, when continuing future research, particular emphasis should be placed on assessing the effect of treatment duration on the therapeutic effect of physiotherapy treatments.

The randomized control study conducted by the authors clearly shows the significant impact of manual therapy procedures on the improvement of the mobility of the temporomandibular joints, reduction of muscle tension and pain in people with TMD. In everyday physiotherapeutic practice, it can be observed more and more often that manual therapy procedures have started to replace physiotherapeutic procedures to a large extent. Despite the higher cost of manual procedures, both therapists and patients observe better therapeutic effects after using MT compared to physical therapy. Therefore, it seems reasonable to implement manual therapy supplemented with physiotherapy treatments in patients with TMD. The conducted studies additionally highlighted the need to continue research on this subject, as the available scientific literature contains a small number of randomized, control studies comparing the effectiveness of physical and manual therapies in patients with TMD. Summing up, as shown by the authors’ clinical experience, the cooperation between the TMD treatment team (including dentist, neurologist, ENT specialist) and a dental physiotherapist should become the ‘gold standard’ in modern dentistry.

As it results from the studies presented above, physiotherapy should be an integral part of the interdisciplinary treatment of patients with painful TMD. While assessing the effectiveness of physical treatments and manual therapy, it was observed that MT is definitely more effective. Manual therapy compared to physical therapy showed a more favourable analgesic (average pain intensity level after the 6th treatment was NRS 0) and myorelaxant effect, thus contributing to the improvement of the mobility of the temporomandibular joints’.

## Data Availability

The datasets used and/or analysed during the current study available from the corresponding author on reasonable request.
